# Open Revolution

**DOI:** 10.1371/journal.pbio.1000078

**Published:** 2009-03-24

**Authors:** Sean R Eddy

## Abstract

Sean Eddy reviews "Opening Up Education," a collection of essays that explores how leaders in the "open education movement" intend to exploit digital communications technology, develop innovative and freely redistributable educational methods and resources, and improve education on a global level.

In 2001, Charles Vest, then President of the Massachusetts Institute of Technology, announced that MIT would make most of its course material freely available online. Browsing the Web site of MIT's Open Courseware (OCW) project (http://ocw.mit.edu), you feel the stirring of a “my God, it's full of stars” transformation: you can borrow material for your courses, study other teachers' teaching methods, maybe even retake college courses you regret having slept through! Remarkably, OCW is just one highly visible part of an “open education movement.” The essays collected in *Opening Up Education*, edited by Toru Iiyoshi and M.S. Vijay Kumar, describe ways in which individuals and institutions intend to exploit digital communications technology, develop innovative and freely redistributable educational methods and resources, and improve education at all levels throughout the world.

But what does “open education” really mean? What is “closed” about education? Should education be free as in no cost, or is there something about education that needs to be freed as in freedom? This sort of ground is already well-trampled by debates about two better-known “open” predecessors, open-source software and open-access publication, and it is instructive to make the comparison.

In software development, almost everyone recognizes the power of sharing, verifying, reusing, and improving source code. At the same time, developing software takes time, which means that it's expensive. It's not obvious that you can give people the right to see, modify, and redistribute your source code without torpedoing your business model. The best open-source advocacy seeks new business models for openly sharing source code without impoverishing software development. The better we reconcile this tension, the better our software will be. The same sort of tension underlies open access. We need computational indexing, searching, and crosslinking of the full text of the scientific literature, but traditional publication business models cannot afford to give open access to full text. The best open-access advocacy promotes innovative publication business models that make full text freely available without putting scientific publishers out of business.

**Figure:**
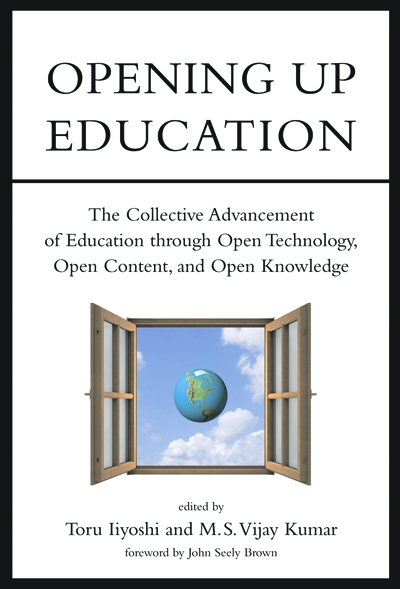


A more utopian “open” advocacy simply denies this real-world tension. Information wants to be free; corporations are evil; people will make great stuff for love not money; free stuff will save the developing world; we'll pay for it with taxes and charity. You don't have to subscribe to Ayn Rand's brand of laissez-faire capitalism to have serious problems with this. It amounts to claiming that intellectual work doesn't take time, or that time isn't worth money—that intellectual property protections exist only to create profit for unnecessary middlemen, not to enable the work of talented professionals who create works that can be readily copied.

So, while I like storming the establishment with pitchforks and torches as much as anyone, when I picked up *Opening Up Education* (or rather, when I downloaded the PDF to my Kindle), I was looking for pragmatism, not utopianism. After 500 pages of “the silos we all know about in higher education are under assault in the new world,” the “hated textbook publishers,” the “epistemological hegemony of higher education,” and the “noble philosophy” of making everything free—“traitors” and “patriots” and “communists,” oh my!—my hopes were beaten down. Many of the 30 essays in this collection are more manifesto than explanation, and many of the 38 authors are writing more for their fellow revolutionary comrades than for us.

The collection's editors—Toru Iiyoshi, a senior scholar at the Carnegie Foundation for the Advancement of Teaching and director of the Knowledge Media Laboratory; and M.S. Vijay Kumar, senior associate dean of undergraduate education and director of the Office of Educational Innovation and Technology at MIT—gathered the authors at a 2006 conference sponsored by the Carnegie Foundation, which is one of the main philanthropic supporters of open-education initiatives. Iiyoshi and Kumar have organized the essays roughly evenly into three sections: Technologies, Content, and Knowledge.

The Technologies essays are mostly about creating open-source software to serve educational purposes. For example, to assist teachers in posting course materials on interactive Web sites, you'd want to deploy some sort of easily customizable course management software in your institution. Several such open-source projects are described, including Bodington (http://www.bodington.org), the Sakai Project (http://sakaiproject.org/portal/), and Moodle (http://moodle.org). The goals of course management overlap with even more ambitious goals of visual learning environments such as Visual Understanding Environment (VUE) (http://vue.tufts.edu), which aim to enable novel visualizations that accelerate learning. One interesting essay described the MIT iLabs project (http://icampus.mit.edu/ilabs/), which aims to make laboratory instrumentation accessible for student experiments via open-source, web-based middleware, standardizing the connection of a professor's laboratory experiments to the Web.

The Knowledge essays are about ways to improve dissemination of teaching methods by enabling better communication among teachers. One of the better essays here, from Randall Bass and Dan Bernstein, points out that professors tend to be isolated from others' teaching experiences (certainly true, in my experience), and they discuss interesting ways to create peer feedback communities. This is all well and good, but there is no tension to resolve with an “open” movement. No one is opposed to better communication, and nothing was really closed by design.

The heart of the book is the Content section, which describes open educational resources. This is where the interesting, real-world tension around educational material and intellectual property restriction arises. We would surely be better off sharing and remixing the best course materials. The trouble is, educational materials are traditionally copyrighted and *protected* from modification and redistribution, rather than being copyrighted and openly licensed using, for example, the groundbreaking Creative Commons licenses (http://creativecommons.org) that specifically *enable* modification and redistribution. Efforts to create and organize openly licensed educational resources are described, ranging from on-line courses (Open University's OpenLearn project, http://www.open.ac.uk/openlearn; Carnegie-Mellon's Open Learning Initiative, http://www.cmu.edu/oli), to freely distributable course material accompanying traditional courses (MIT Open CourseWare, http://ocw.mit.edu), to collections of smaller modules that can be remixed by educators (MERLOT, http://www.merlot.org; Connexions, http://www.cnx.org). It is an extraordinary and ambitious set of efforts, all well worth knowing about.

Nonetheless, when I actually went to these sites, it became clear how far they have to go before they can compete with a good book. Too many resources I saw were sketchy, incomplete, and unsatisfying—more akin to Peter Norvig's version of the Gettysburg Address than Abraham Lincoln's original (Norvig's is a wicked, content-free PowerPoint satire with bullet points: “shared vision,” “what makes nation unique”). In his essay, Stuart Lee, director of computing services at Oxford University, touches on a key insight. Distributing open-source software or open-access literature is only a matter of attaching an open license to a finished product, but most of an educator's course materials are rarely a finished, free-standing work. Course materials are more usually fragmentary, cobbled-together *aide-mémoires* that only make sense in the context of face time in the course. A lot of work must go into each piece of content to raise it to the quality of textbook material, and yet more work is required to have the material best use the interactive capabilities of the Web. The most impressive content was the least ambitious, capturing and distributing the existing output of traditional courses in new ways—YouTube videos of MIT lectures, for example.

The disparity between substance and vision was addressed best in two of the collection's more sober essays. Clifford Lynch, director of the Coalition for Networked Information, leads his essay with a 150-year-old quote from Thomas Carlyle, “the true university today is a collection of books.” Lynch is almost alone here in recognizing that books and public libraries already “open” education to a great extent. He writes a well-considered examination of how digital resources can build and improve on the time-tested foundation of books and libraries we already have. Diane Harley, an anthropologist at Berkeley, has actually studied how teachers use and remix materials in their courses. Her scholarly and data-driven essay includes many insights, including pointed warnings to the digerati:

*The chasm between what many technological enthusiasts envision in terms of scale and quality of use on one hand, and what productive and creative academic scholars say they need on the other, is manifested in the suggestion that “the lack of willingness of faculty to change” is a key barrier to wider adoption of and demand for a variety of technologies, and digital content in scholarship.*

“Remix,” “collective wisdom,” “Web 2.0”—many of these essays ride a bubble of popular digital punditry enthusiastically but too uncritically. Many technologists today are infected with an idea that “community is king,” that high-quality content will rain down freely merely because we connect digital communities openly. This confuses ways of *sharing* ideas with ways of *creating* ideas. It is a kind of magical thinking that has much in common with the cargo cults that cut landing strips in the jungle and carved radios from sticks in hope that more sophisticated beings would parachute technological artifacts down upon them. With all respect to the passionate and pioneering initiatives described in this collection, building landing strips to receive open educational content will not be enough. More attention must be paid to the fact that someone still needs to spend time painstakingly developing artful ways to make difficult concepts understandable—to teach!—and that it will take even *more* time (thus money) to render these hard-won ideas using multimedia web technology compared with writing textbooks. Success hinges on the adoption of open licensing by the professionals who make digital educational resources, and on finding ways to finance their work.

